# Neuron/Glial Antigen 2-Type VI Collagen Interactions During Murine Temporomandibular Joint Osteoarthritis

**DOI:** 10.1038/s41598-018-37028-1

**Published:** 2019-01-11

**Authors:** Mamoru Yotsuya, Andrew E. Bertagna, Nageeb Hasan, Scott Bicknell, Toru Sato, David A. Reed

**Affiliations:** 1 University of Illinois at Chicago, Department of Oral Biology, Chicago, IL USA; 2 Tokyo Dental College, Department of Fixed Prosthodontics, Tokyo, Japan

## Abstract

The degeneration of articular cartilage underscores the clinical pathology of temporomandibular joint osteoarthritis (TMJ-OA) and is promoted through dysfunctional biochemical or biophysical signaling. Transduction of these signals has a multifaceted regulation that includes important cell-matrix derived interactions. The matrix encapsulating the cells of the mandibular condylar cartilage (MCC) is rich in type VI collagen. Neuron/glia antigen 2 (NG2) is a type I transmembrane proteoglycan that binds with type VI collagen. This study defines the temporospatial dynamics of NG2-type VI collagen interactions during the progression of TMJ-OA. Membrane-bound NG2 is found to colocalize with pericellular type VI collagen in superficial layer cells in the MCC perichondrium but is present at high levels in the cytosol of chondroblastic and hypertrophic cells. When TMJ -OA is induced using a surgical instability model, localized disruptions of pericellular type VI collagen are observed on the central and medial MCC and are associated with significantly higher levels of cytosolic NG2. NG2 localized within the cytosol is found to be transported through clathrin and dynamin mediated endocytic pathways. These findings are consistent with NG2 behavior in other injury models and underscore the potential of NG2 as an entirely novel molecular mechanism of chondrocyte function contextually linked with TMJ-OA.

## Introduction

Temporomandibular joint osteoarthritis (TMJ-OA) is a clinical syndrome of arthralgia, limited joint mobility, and diminished quality of life^1,2^. Contemporary molecular targets for clinical intervention have yet to be determined due in part to an unknown etiology and ill-defined pathophysiology. Risk factors for TMJ-OA are complex, and contextually linked with inflammatory, metabolic, and mechanical stresses in the joint. Pathogenic biochemical and biophysical stimuli are hypothesized to be transduced to cells through a distinct division of the extracellular matrix encapsulating the chondrocytes called the pericellular matrix (PCM)^3–6^. In the mandibular condylar cartilage (MCC), the PCM is comprised primarily of type VI collagen, type IV collagen, and laminin^7^. The chondrocyte anchors to type VI collagen^8^ through β1-integrin and neuron-glia antigen 2 (NG2), also known as Chondroitin Sulfate Proteoglycan 4 (CSPG4)^9,10^. NG2 is type I transmembrane proteoglycan expressed in a range of cells including oligodendrocyte progenitor cells, smooth muscle cells, fibroblasts, pericytes, and chondroblasts.

NG2 is a multifunctional, multidomain molecule that broadly influences cell migration and proliferation^11^, and may potentiate the cell’s response to injury^12–16^. The N-terminal ectodomain has three functionally distinct subdomains^17^. Subdomain 1 on the amino-terminal consists of a globular confirmation stabilized through disulfide bonding. The central subdomain 2 contains chondroitin sulfate chains, binding sites for the mitogenic molecules Basic Fibroblast Growth Factor (bFGF; FGF2) and Platelet Derived Growth Factor (PDGF)^18^, and a type V and VI collagen binding domain^9,10,19^. Subdomain 3 contains two sites for proteolysis and shedding of the ectodomain^20^. Ligand binding and ectodomain shedding are hypothesized to promote C-terminal intracellular domain interactions with multiple signaling pathway molecules including extracellular signal-regulated kinases 1/2 (ERK) and protein kinase C-alpha (PKC-α), and through a PDZ binding motif. The intracellular domain can further modify NG2 functionality through Thr^2256^ and Thr^2314^ phosphorylation^11^.

Alteration in the localization of NG2 on the cell surface is associated with cell stress response. In periodontal ligament fibroblasts, elevated levels of membrane-bound NG2 propagate anoikis while protease mediated cleavage of the ectodomain attenuates anoikis^16^. In tendon fibroblasts, mechanical strain injury lowers cell surface expression levels of NG2, disrupting colocalization with type VI collagen^13^. Membrane-bound NG2 can be proteolytically shed to activate translocation of the intracellular domain into the cytoplasm^21^, potentially promoting cell cycle withdrawal^13,22^. In oligodendrocyte precursor cells, this translocation into the cytosol regulates oxidative stress pathways through NG2 sequestration of OMI/HtrA2 in the caspase 3 regulatory pathway^14^.

Domain-specific NG2 functionality in chondroblasts has yet to be fully resolved. NG2 is expressed in limb growth-plate cartilage^23^, articular cartilage^24^ and in chondrosarcomas^25^. In osteoarthritic chondrocytes, NG2 adhesion with type VI collagen is compromised^24,26^. NG2 in secondary cartilages such as the TMJ has never been characterized. NG2 has promise as a critical regulator of TMJ cartilage homeostasis because of a pericellular matrix rich in type VI collagen^7^, the presence of the NG2 ligands FGF2 and PDGF^27,28^, and the elevated levels of NG2 active proteases MMP13, MMP14, and ADAM10 during TMJ OA^29,30^. The goal of this study was to evaluate and define the interaction of the NG2 ectodomain with type VI collagen during the progression of TMJ OA and to evaluate if NG2 internalization is contextually linked with the inflammatory and/or mechanical stresses associated with TMJ OA.

## Results

### NG2 colocalizes with pericellular type VI collagen in the MCC

**Figure 1:**
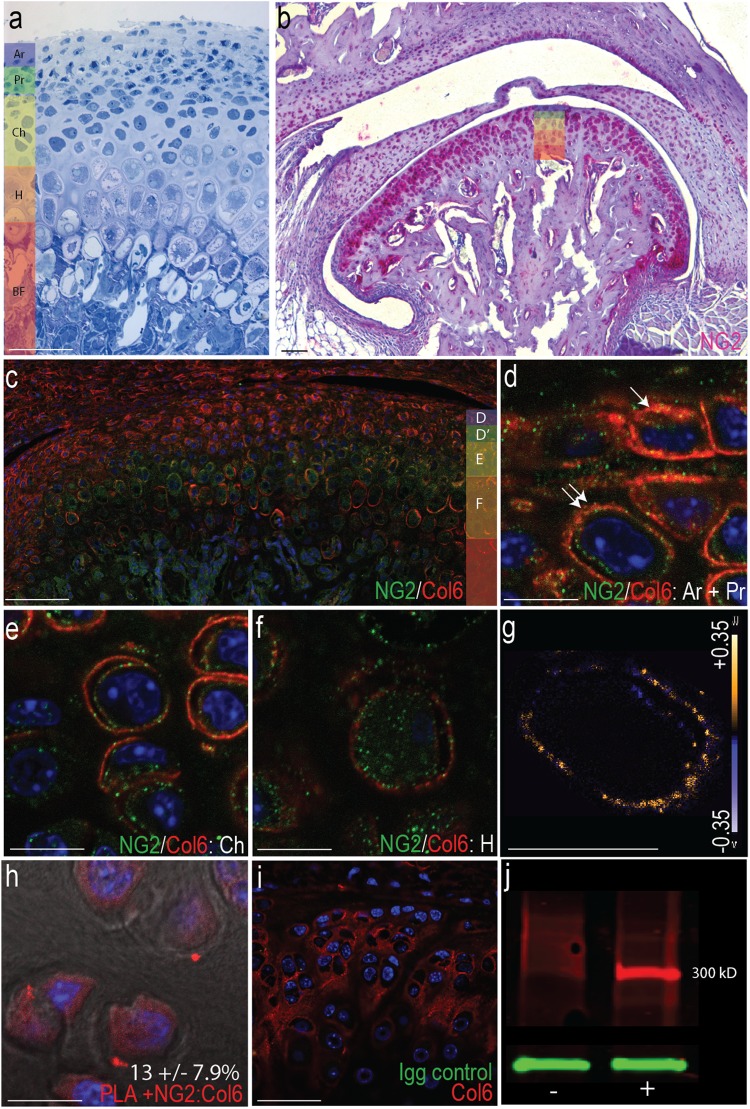
Distribution of NG2 in the cartilage of the temporomandibular joint. (**a**) histomorphometric delineations of four cell populations in the mature mandibular condylar cartilage (MCC) including the articular (Ar), proliferative (Pr), chondroblastic (Ch) and hypertrophic (H) cells located superficially to the zone of bone formation (BF). (**b**) distribution of NG2 in MCC using a rabbit polyclonal antibody illustrating that all cells stain positive for NG2. Cell layers are color coded (**c**) Colocalization of NG2 (green) and type VI collage (red) in non-surgical control tissue. In Ar, Pr, and Ch cells, NG2 is membrane bound. In H cells, NG2 is localized to the cytosol. Magnified images in d-f illustrate NG2-type VI collagen interactions in each cell layer, defined with the color-coded bar to the right (**d**) articular (single arrow) and proliferative (double arrow) cells (**e**) chondroblastic cells and (**f**) hypertrophic cells. (**g**) the colocalization of membrane-bound NG2 and pericellular type VI collagen in the Pr cell indicated in d visualized with a PDM plot, with high overlap indicated by yellow and no overlap in blue (**h**) colocalization of NG2-type VI collagen from a proximity ligation assay, with the average percentage of PLA positive cells reported in the lower right (n = 3) (**i**) a secondary antibody Igg control showing no non-specific staining.) (**j**) western blot of NG2 from primary mandibular chondrocytes without (−) and with (+) a 1-hour Chondroitinase ABC treatment. Scale bars a-c and i equal 50 µm. Scale bars d-h equal 10 µm.

To investigate the spatial distribution of NG2 and pericellular type VI collagen in the mandibular condylar cartilage (MCC), we immunolabeled 16–20-week-old tissue from non-surgical control mice. NG2-type VI collagen interactions were evaluated in each functionally distinct cell layer of the MCC (defined in Fig. 1a). NG2 was found in nearly all MCC cells, at especially high density on the lateral and medial margins (Fig. 1b). Immunofluorescent labeling of NG2 and type VI collagen illustrates a heterogeneous distribution in the MCC (Fig. 1c). High levels of membrane-bound NG2 were observed in articular, proliferative, and some chondroblastic cells. Type VI collagen was organized into tight pericellular bands (Fig. 1d,e) and NG2/type VI collagen colocalization was verified by a measure of colocalization, the Mander’s Overlap Coefficients (Fig. 1g). Some NG2 was observed in the cytosol at low levels. Higher levels of cytosolic NG2 were observed in hypertrophic cells, corresponding with diminished pericellular type VI collagen (Fig. 1f). Colocalization was validated using proximity ligation assay with positive signal in articular and proliferative cells and  13 (±7.9)% of these cells PLA positive (Fig. 1h). Western analysis of cultured mandibular chondrocytes using NG2 antibody illustrates a single band at 300 kD, indicating membrane-bound NG2 is likely present in its full length form in the MCC^20^. Chondroitinase ABC treatment illustrates that the subdomain 2 chondroitin sulfate chain is present on membrane-bound NG2 in mandibular chondrocytes (Fig. 1j).

### Degeneration results in localized disruption of the pericellular matrix

**Figure 2:**
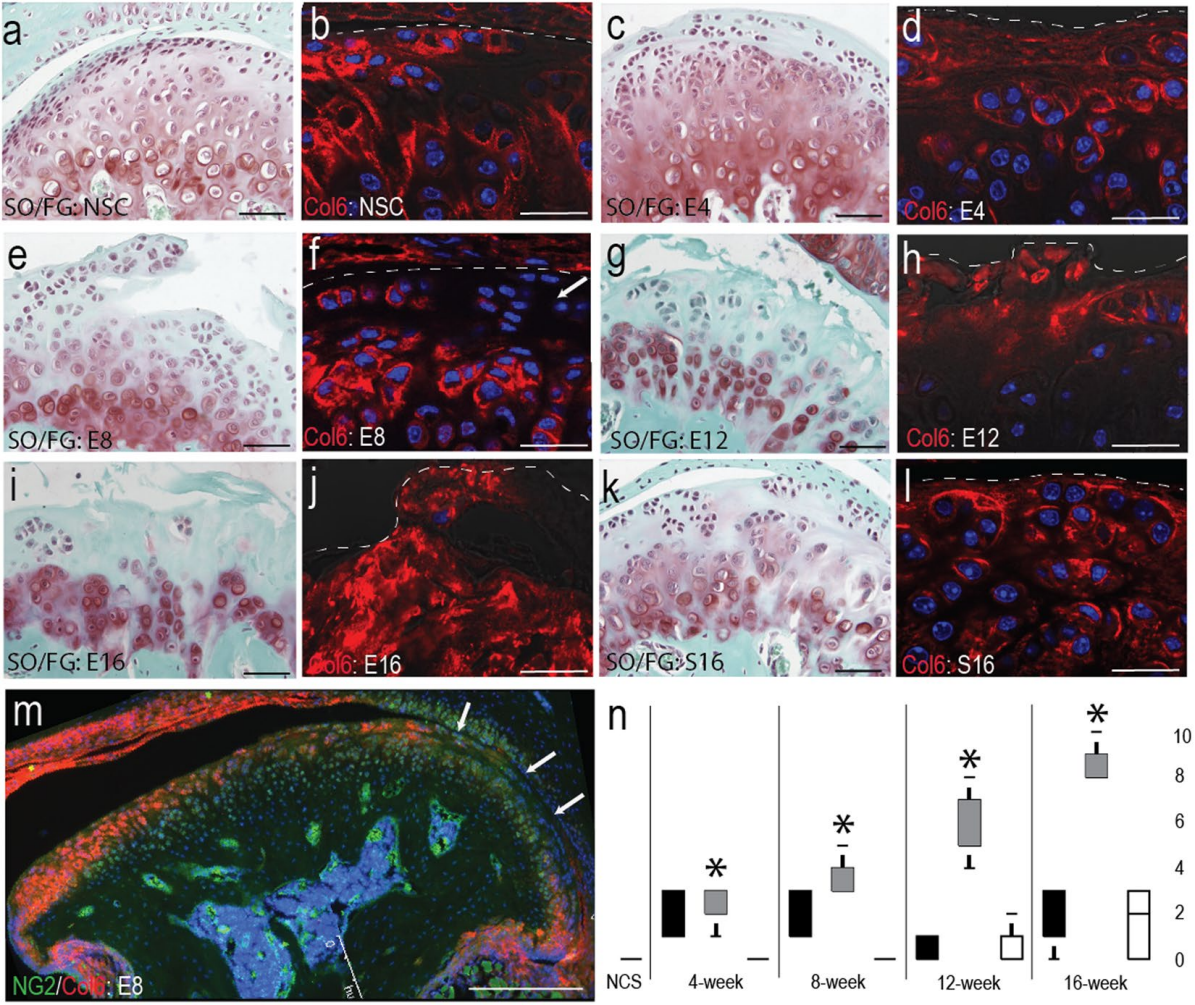
Induction of degenerative joint disease through unilateral partial discectomy. Safranin-O/Fast Green (SO/FG) staining and type VI collagen (Col6) immunolabeling ipsilateral to discectomy in non-surgical control (**a**,**b**) and post-surgical discectomy after four (**c**,**d**), eight (**e**,**f**), twelve (**g**,**h**), and sixteen (**i**,**j**) weeks and 16 week post-surgical sham controls (**k-l**). Note the lack of pericellular type VI collagen in superficial layer cells at 8 week post-surigical (arrow) m) immunofluorescent staining of type VI collagen in an 8-week post-surgical MCC illustrating the loss of pericellular type VI collagen in superficial layer cells on the medial joint (arrows). n) pathology graded using Modified Mankin Scoring of ipsilateral sham joints (black) ipsilateral discectomy joint (grey) and contralateral discectomy joints (white) at each time point. Statistical significance was determined using by a t-test assuming unequal variances and indicated with an asterisk (n = 4). All sections for Mankin Scoring were selected from the middle condyle. Differences in the appearance of the condyle are the result of variation in condylar flattening and bony remodeling. Medial is to the right in all images. Scale bars a, c, e, g, i, k equal 50 µm. Scale bars b, d, f, h, j, l equal 25 µm. Scale bar for equals 250 µm.

To evaluate temporospatial changes in NG2 and type VI collagen during the progression temporomandibular joint osteoarthritis (TMJ-OA), we utilized a unilateral partial discectomy surgical instability murine model. Compared to non-surgical and sham controls (Fig. 2a,b,k,l), 4-week post-surgical tissue presented with condylar flattening and lipping, an increase in background proteoglycan, a thickened fibrous layer on the articular surface (Fig. 2c). Type VI collagen was present in the interterritorial regions between cells in the thickened superficial layer (Fig. 2d). At 8-weeks post-surgical, chondral lesions and fibrillation began to form in the articular layer with a decrease in background proteoglycan. Pericellular type VI collagen appeared locally diminished in articular and proliferative cells on the central and medial MCC (Fig. 2e,f). At 12-weeks post-surgically, lesions and fibrillations extend beyond the articular layer, background proteoglycan was lower, pericellular proteoglycan was elevated, and hypocellularity in the articular layer was evident (Fig. 2g). Large regions devoid of cells and type VI collagen are evident at this time point (Fig. 2h). At 16-weeks post-surgical, the articular layer is almost devoid of cells and proteoglycans (Fig. 2i) and type VI collagen strongly stains on the lateral and medial margins but not the center of the superficial layer of the MCC (Fig. 2j). The sham control presents with some cell clustering and lower background proteoglycans (Fig. 2k) but type VI collagen that is normal in its distribution in the pericellular matrix (Fig. 2l). Quantification of degeneration with a Modified Mankin Score illustrates that the MCC ipsilateral to the surgery has a significantly higher score at all time points compared to the non-surgical and sham control at all time points (Fig. 1n).

### Degeneration promotes NG2 internalization in articular chondrocytes

**Figure 3:**
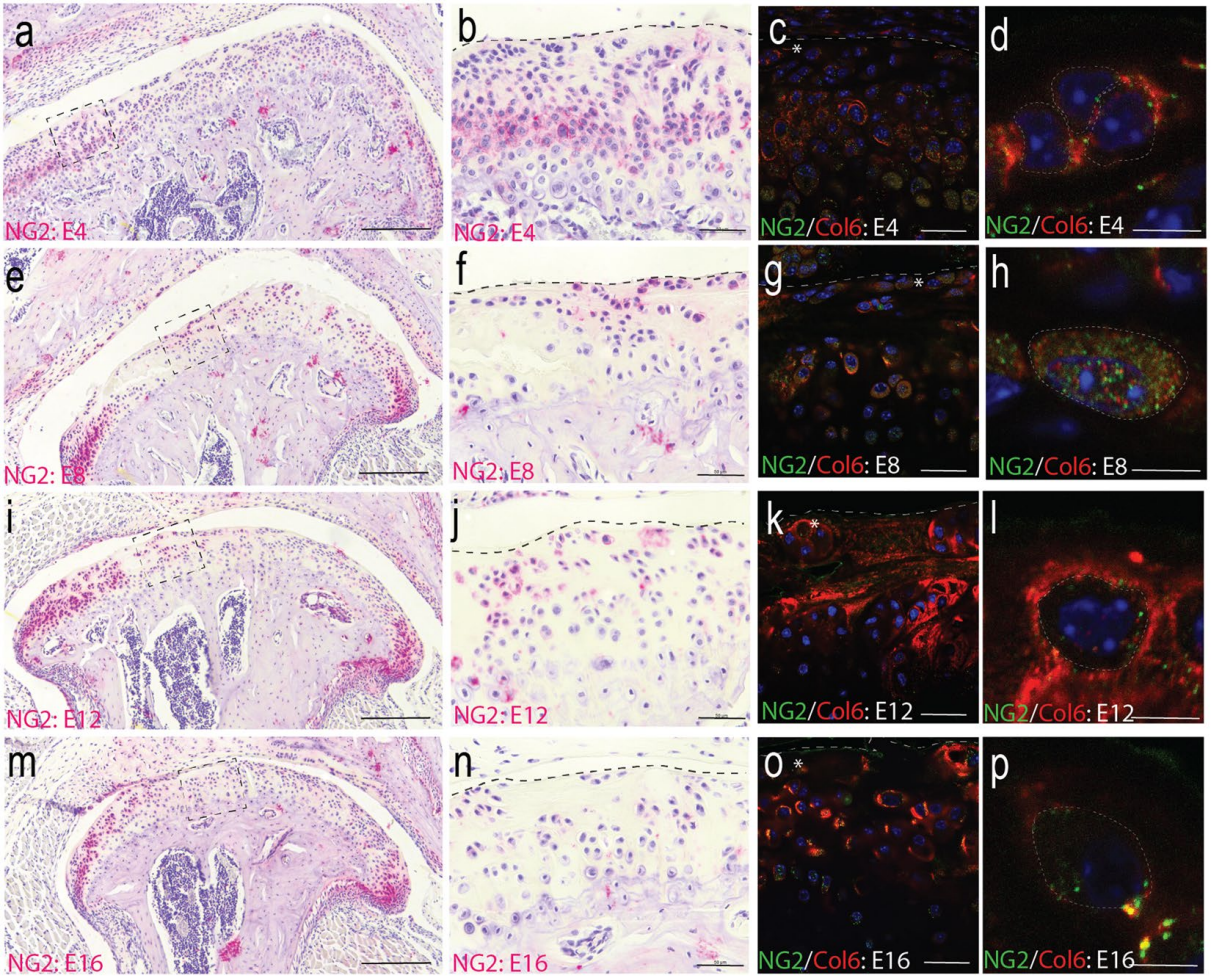
The spatial distribution of NG2 during the progression of TMJ-OA. (**a**,**b**) at 4 weeks post-surgical, NG2 immunolabeling illustrates lower staining in the superficial layer of the cartilage at the center of the condyle but strong staining at the lateral and medial margins of the MCC. (**b**,**c**) NG2-type VI collagen immunofluorescence illustrates that the NG2 present in articular cells on the center condyle is mostly membrane bound. (**e**,**f**) at 8-weeks post-surgical, NG2 staining has increased in the articular layer cells on the center of the condyle. (**g**,**h**) NG2-type VI collagen immunofluorescence illustrates that in regions where pericellular type VI collagen is diminished, NG2 is present at high levels and has a punctate appearance inside of the cytosol. (**i**,**j**) at 12-weeks post-surgical, there are fewer cells in the articular layer of the condyle and NG2 staining is low in in these remaining cells. (**k**,**l**) NG2-type VI collagen immunofluorescence illustrates a spatial distribution of NG2 similar to that of the non-surgical control. (**m**–**p**) at 16-weeks post-surgical, the articular layer is mostly hypocellular. The remaining cells stain with low levels of NG2 and type VI collagen. All sections taken from the center of the condyle. Differences in the gross morphology reflect differences in condylar flattening and bony remodeling associated with the murine model. Medial is to the right in all images. Scale bar a, e, i, m is equal 200 µm. Scale bar b, f, j, n equal 50 µm. Scale bars for c-d, g-h, k-l, o-p equal 10 µm.

**Figure 4:**
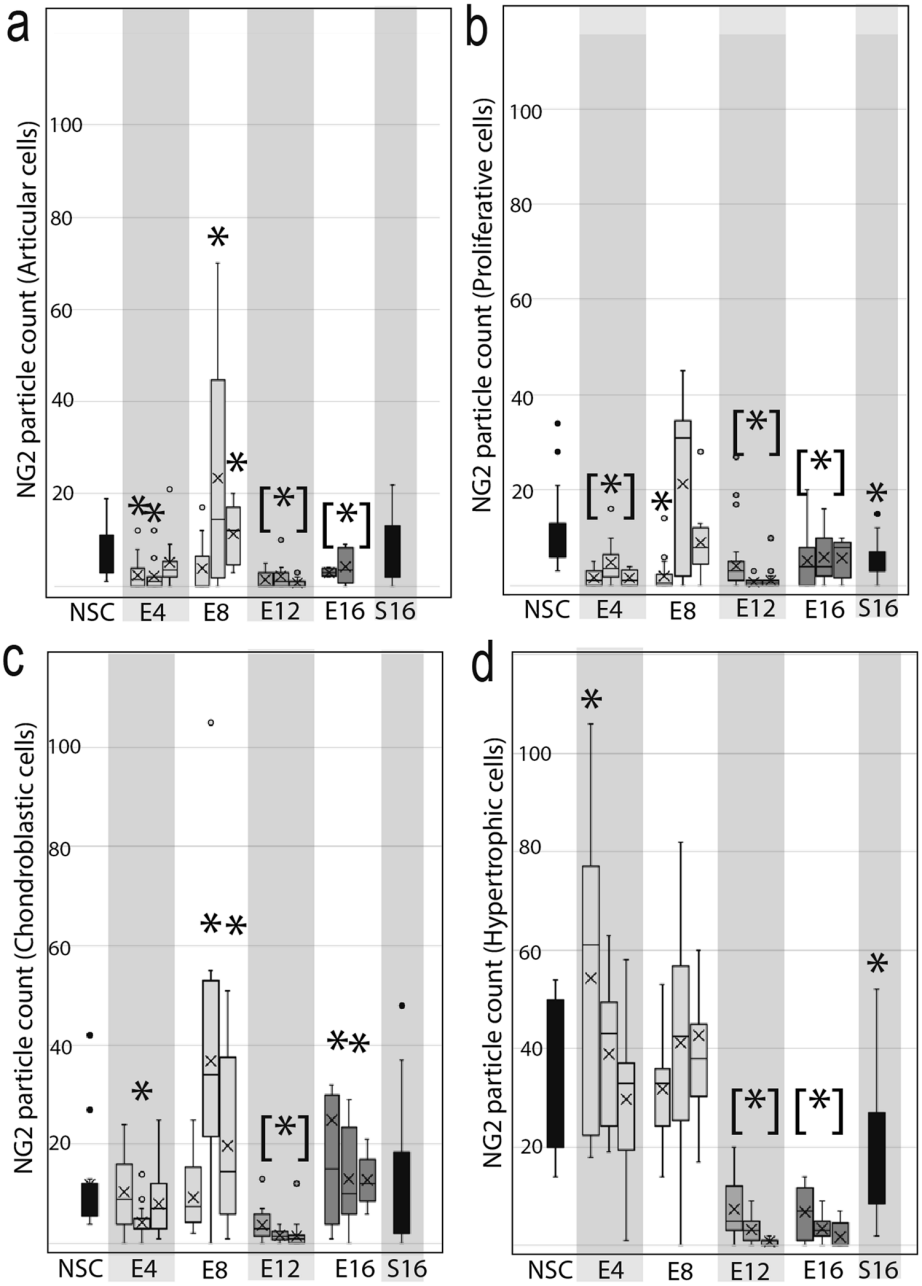
Quantification of punctuate NG2 particles in TMJ OA cells. The number of punctate NG2 particles within the cytosol was quantified from confocal immunofluorescent images of NG2-type VI collagen from each experimental group and compared to non-surgical and sham control mice (n = 3). NG2 particle count was calculated from five cells in each tissue layer including the articular (**a**), proliferative (**b**), chondroblastic (**c**), and hypertrophic (**d**) cells. For each experimental group, particle count was quantified in three regions of condyle with measurements from the lateral condyle in the left box, center condyle in the center, and medial condyle to the right. There were no significant regional differences for the non-surgical and sham controls so the data are pooled. Statistical significance to the non-surgical control was determined by a t-test assuming unequal variances and indicated by asterisks.

To evaluate temporospatial changes in NG2 during TMJ-OA, discectomy tissue was immunolabeled at each experimental timepoint. At 4-weeks post-surgically, the superficial layer cells have low expression of NG2 (Fig. 3a) compared with non-surgical controls (Fig. 1b). At 8 and 12-weeks post-surgically, superficial layer cells begin to form NG2 positive clusters (Fig. 3e,f,i,j). 16-weeks post-surgically, there is high hypocellularity and few NG2 positive cells (Fig. 3m,n). To evaluate NG2-type VI collagen interactions during TMJ-OA, epitopes were immunofluorescently labeled. At 4 weeks post-surgically, articular and proliferative cells were associated with membrane-bound NG2 and hypertrophic cells with cytosolic NG2 (Fig. 3c,d). At 8-weeks post-surgically, the level of cytosolic NG2 increased in all cells in the superficial layer forming a punctate appearance within the cytosol (Fig. 3g,h). At 12 and 16-weeks post-surgically, all superficial layer cells had low levels of membrane-bound and cytosolic NG2 (Fig. 3k,l,o,p). To calculate the amount of NG2 in the cytosol, the punctate cytosolic particles of NG2 were quantified from a region of interest defined by inside of the pericellular matrix. The analysis was grouped into three spatial regions to account for regional variations across the MCC. In articular and proliferative cells, lower levels of cytosolic NG2 were found at 4-weeks post-surgically (Fig. 4a,b). Cytosolic levels increased in articular and chondroblastic cells at 8-weeks post-surgically in the center and lateral MCC (Fig. 4a,c). Hypertrophic cells were found to have the highest number of punctate NG2 in non-surgical controls tissues which significantly decreased by 12-weeks post-surgical (Fig. 4d).

### Internalized NG2 is associated with clathrin and dynamin mediated endocytosis

**Figure 5:**
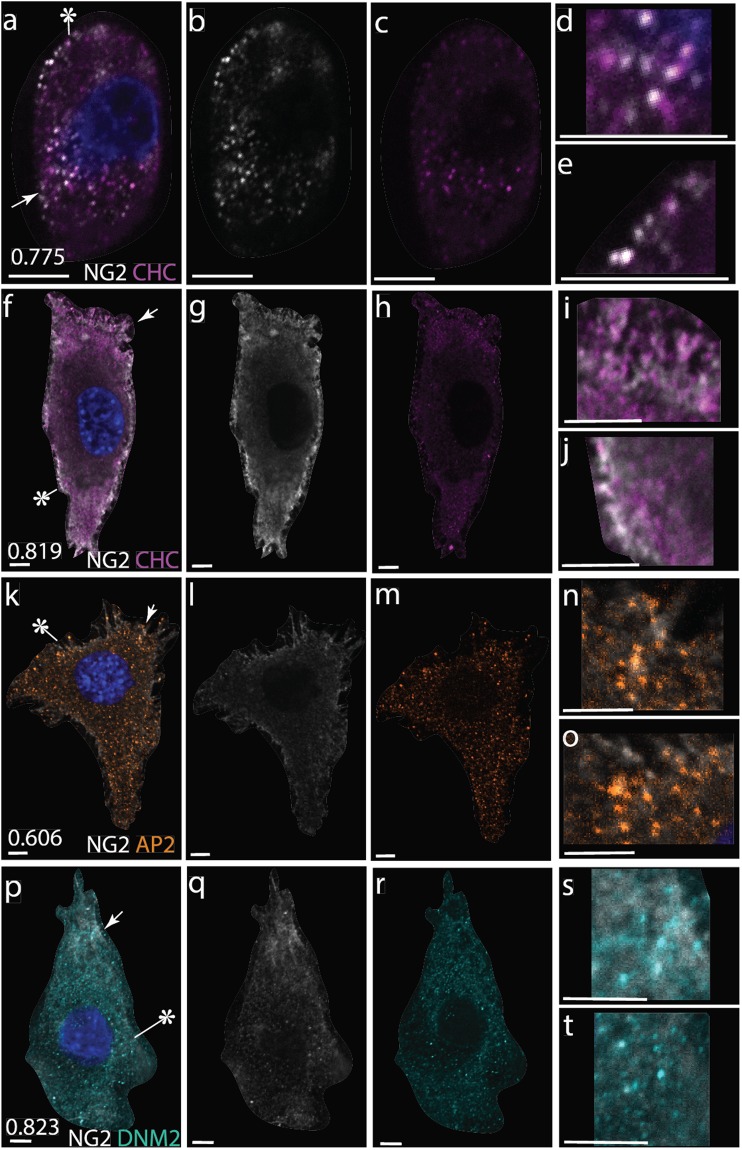
Confocal immunofluorescence illustrating colocalization of NG2 (white) with markers for endocytosis in the chondrocyte cytosol. (**a**–**e**) High levels of overlap between NG2 (mouse, monoclonal) and clathrin heavy chain (CHC; Rabbit, monoclonal) are observed *in vivo* particularly in hypertrophic cells with high levels of cytosolic NG2. In a, the arrow indicates the upper expanded region in d, asterisk indicates expanded region in e and the number in lower left corner is the amount of colocalization reported as a Mander’s overlap coefficient defined as the sum of the products of the two channels absolute intensities divided by the maximal product, corresponding to perfect colocalization. *In vitro* primary mandibular chondrocyte monolayer cell cultures illustrate a similar co-expression patterns with NG2 (Rabbit, polyclonal) and (**f**–**j**) CHC (mouse, monoclonal;), (**k**–**o**) α-adaptin (AP2, mouse, monoclonal), and (**p**–**t**) dynamin II (DNM2, mouse, monoclonal). Note that NG2 colocalization with endocytic markers are concentrated primarily near the cell walls. All scale bars equal 5 µm.

The punctate NG2 appearance inside of the cytosol could be attributed to multiple causes including proteolysis of the NG2 ectodomain, imaging artifact, and active transport of the protein by the cell. To assess if NG2 was being actively transported into the cell during TMJ OA, we immunolabeled primary condylar chondrocytes and TMJ sections with NG2 and markers of clathrin mediated endocytosis including α-adaptin (AP2), dynamin II (DNM2), and clathrin heavy chain (CHC). The pericellular matrix was delineated by type VI collagen and Z-stacks were used to distinguish membrane-bound and cytosolic NG2. Cytosolic NG2 was found to overlap with CHC *in vivo* and CHC, AP2, and DNM *in vitro*. NG2-endocytosis markers were concentrated near the cell membrane and typically concentrated near one pole of the cell however not all cytosolic NG2 was found to colocalize with endocytic markers (Fig. 5).

## Discussion

This study focuses on the temporospatial dynamics of chondrocyte-pericellular matrix (PCM) interactions in temporomandibular joint osteoarthritis (TMJ-OA). The PCM both influences and regulates the biochemical and biophysical cellular microenvironment and is an established mediator of tissue homeostasis and dysfunction^4,7,31^. The PCM of mandibular condylar cartilage (MCC) is composed of primarily type VI collagen. Neuron/glia antigen 2 (NG2) is a transmembrane proteoglycan that has not been previously describe in the TMJ, but is known to interact with type VI collagen^9^.

Membrane-bound NG2 colocalizes with type VI collagen in the pericellular matrix of the superficial layer cells of the perichondrium (Fig. 1d). In the deeper layers of the MCC, hypertrophic cells were found to have high levels of cytosolic NG2 and lower levels of pericellular type VI collagen (Figs 1f and 4d). This heterogenous pattern of NG2-type VI collagen interactions in the MCC may reflect functional differences in the cell population that confer on the tissue unique growth dynamics that are dependent upon the coordinated migration, proliferation, and differentiation of the cells in the perichondrium. The superficial layer of the perichondrium constitutes the articular surface and is continuous with the fibrous layer of the bony periosteum. The deeper layer of the perichondrium consists of undifferentiated prechondroblastic cells continuous with the osteogenic layer of the periosteum that proliferate and differentiate to influence chondrogenesis and osteogenesis at the condyle^32^. Exposing mandibular chondrocytes to type VI collagen promotes proliferation^7,33^. Conversely, blocking NG2 inhibits migration toward type VI collagen^19^, lowers membrane associated type VI collagen, and promotes cell cycle withdraw in the presence of mechanical stress^13^. Thus, there is strong support for the hypothesis that NG2-type VI collagen interactions in the MCC perichondrium potentiate a proliferative phenotype in the undifferentiated prechondroblastic layer.

When TMJ -OA is induced using a surgical instability model, localized disruption of pericellular type VI collagen is observed on the central and medial MCC. By 8-weeks post-surgical, superficial layer cells are found to have significantly higher levels of internalized NG2. Superficial layer hypocellularity then increases at 12 and 16 weeks post-surgically, with membrane-bound NG2 expressed only at cell clusters. These changes were concentrated on the medial and central regions of the condyle, opposite the presentation observed in the human TMJ-OA^34^. Changes localized to one region of the condyle underscore the importance of biophysical stress as a mediator of degeneration^35^. Therefore, we believe that the differences observed from the clinical presentation represent contact stresses associated with the animal and surgical model.

Changes in pericellular matrix organization and composition can alter the biophysical and biochemical microenvironment of the chondrocyte. In limb cartilage, decreases in type VI collagen lower the stiffness of the pericellular matrix^4^. *In vitro*, mechanical loading of limb chondrocytes releases perlecan sequestered FGF2 and promotes ERK activation^31^. Therefore, the observed changes in pericellular type VI collagen may promote the progression of TMJ-OA through biophysical or biochemical pathways. NG2 is a promising candidate for signaled dysfunction in the PCM not only because of the colocalization with type VI collagen but also because of known high affinity biding with FGF-2 and PDGF-aa^18^.

Alterations in the localization of NG2 are observed together with alterations in pericellular type VI collagen organization. A significant increase in cytosolic NG2 is observed in superficial layer articular cells at 8-weeks post-surgically, followed by hypocellularity and clustering of NG2 positive cells at 12 and 16 weeks post-surgical. This observation is consistent with NG2 behavior in other injury models. Proteolytic shedding of the ectodomain lowers membrane-bound NG2 and attenuates apoptosis^16^ potentially through inhibition of oxidative stress pathways^14^. The internalized NG2 observed in TMJ OA may be acting in a similar manner to sequester and inhibit upstream regulators of apoptosis or anoikis. However, we were unable to satisfactorily extract and purify NG2 from healthy and diseased murine MCC tissue. Quantifying *in vivo* NG2 shedding in TMJ-OA tissue is an important next step.

We identify one potential pathway of NG2 internalization, with at least some of the cytosolic NG2 colocalizing with clathrin heavy chain both *in vivo* and *in vitro. In vitro*, cytosolic NG2 also colocalizes with α-adaptin and dynamin II, consistent with other previous reports^36^. Clathrin and dynamin mediated endocytosis is associated with lysosomal activity and plays an important role in ligand-receptor recycling. The mitogenic ligands FGF2 and PDGF-aa and their receptors are known to be closely associated with full length, membrane-bound NG2^18^ and are both present in mandibular condylar cartilage^28^. Future work will focus on defining the association of NG2 with ligand-receptor complexes recycled back to the membrane or sent to lysosomes of degradation. This mechanism would contextually link NG2 dynamics with the attenuation or termination of key TMJ-OA associated ligand-receptor signaling^37^.

## Methods

### Surgical instability mouse model

TMJ-OA was induced by unilateral partial discectomy following Xu *et al*. (2009). Seven-week-old, male c57 BL/6 mice were anesthetized with ketamine (100 mg/kg) and xylazine (5 mg/kg). The skin over the TMJ was shaved and cleaned with 70% ethanol and betadine. A 1 mm incision was made over the temporomandibular joint (TMJ), the lateral capsule was exposed, the articular disc excised, the joint was irrigated, and closed with 5–0 Ethilon suture. Sham control surgeries were identical except the lateral capsule and disc remained intact. Experimental endpoints included four time points: 4, 8, 12, and 16 weeks post-surgical, and data are compared with 16-week sham and age matched non-surgical control mice. All experiments using vertebrate animals were approved by the University of Illinois at Chicago Animal Care Committee and performed in accordance with the relevant guidelines and regulations.

### Grading TMJ degeneration

Tissue samples were fixed in 4% PFA for 18–24 hours, decalcified with 4.5% EDTA for 28 days, and paraffin embedded. Tissue blocks were serially sectioned at 8 µm. For histomorphometric analysis, sections from the middle of the condyle were selected, deparaffinized, and stained with safranin-o and fast green. A sample size of four mice were used for each experimental group. There were slight differences in the gross morphology of the post-surgical condyles resulting from variations in condylar flattening and bony remodeling. However, the patterns of cartilage degeneration were similar in all cases. The progression of TMJ-OA at each experimental time point was quantified using a Modified Mankin Score following Xu *et al*. (2009). Modified Mankin scores were statistically compared using a t-test assuming unequal variance

### Primary cell culture of mandibular condylar chondrocytes

Primary culture follows published methods for chondrocytes^38,39^. In short, MCC was collected from 10–20 day old mice in CO2 independent collection medium (Gibco, 18045–088), washed in 1x sterile PBS supplemented with 25 mg/ml Plasmocin (InVivoGen, ant-mmp), 50 U/ml penicillin and 0.05 mg/ml streptomycin (Sigm, P0781) for 5 minutes, transferred to a 3 mg/ml type II collagenase (Worthington Biochemical, LS004174) in DMEM (Gibco, 11966–025) digestion medium for one hour, and then transferred to a 1 mg/ml collagenase digestion medium overnight in 5% CO2 at 37 °C. All cell medium and washes were supplemented with 25 mg/ml Plasmocin (InVivoGen, ant-mmp), 50 U/ml penicillin and 0.05 mg/ml streptomycin (Sigm, P0781), and 2mM _L_-Gln (Sigma, G7513). After digestion, cells were dispersed by agitation with a serological pipette, filtered through a 40 µm cell strainer, centrifuged at 10,000 g for 15 minutes at room temperature, the pellet was re-suspended in supplemented sterile 1x PSB, and centrifuged again. The resulting pellet was re-suspended in 10 ml of FBS supplemented advanced DMEM (Gibco 12492–013). Cell density was calculated with a hemocytometer, and cells were plated at 10 × 10^4^ cells/cm^2^ with a glass cover slip. Culture medium was replaced every two days until confluence at approximately 8 days.

### Western blot

Cells were washed in ice cold sterile 1x PBS and lysed with ice cold NP40 lysis buffer (Invitrogen) supplemented with Halt Protease Inhibitor Cocktail (Thermo Fisher) for 3 hours. Lysate insolubles were removed by centrifugation at 14000 g for 15 minutes at 4 °C. Total protein concentration was determined by Bradford Assay. The supernatant was incubated with and without Chondroitinase ABC (AMSBio, 100330-1 A) for 3 hours at 37 °C. Lysates were adjusted to a 1x Protein Sample Loading Buffer (Licor, 928–40004), heated at 100 °C 5 minutes, run on a 4–15% sodium dodecyl sulfate polyacrylamide gel (SDS-PAGE), and analyzed by Western Blot with antibodies against a rabbit polyclonal NG2 (Millipore, MAB 5320) and a β-actin mouse monoclonal control (Licor, 926–42210). A sample size of 3 was used for all western blots.

### Immunohistochemistry and immunocytochemistry

Chromogen immunolabeling was performed with a multiplex mouse-HRP/rabbit-AP IHC kit (Enzo, MULTIVIEW Plus, ENZ-KIT181-0150). For immunofluorescence, sections were pre-treated with Collagenase D (3 mg/ml) and Hyaluronidase (2 mg/ml), permeabilized with methanol and 0.5% (v/v) Triton. Antigen retrieval included two 30-minute sodium borohydride (5 mg/ml) washes and a 0.01 M citrate buffer wash at 95 °C for 10 minutes (pH 6). Tissue was blocked in 5% donkey serum (Sigma D9663) and primary antibody incubations were overnight at 4 °C. Primary antibodies include NG2 rabbit polyclonal (Millipore, MAB5320), NG2 mouse monoclonal (Millipore, MAB5384; clone 132.39), Collagen 6α1 rabbit polyclonal (Fitzgerald, 70R-CR009x), clathrin heavy chain rabbit monoclonal (CHC, Cell Signaling, D3c6 XP4796), α-adaptin mouse monoclonal (AP2, Invitrogen, MA1-064), and dynamin II mouse monoclonal (DNM2, BD Biosciences, 610263). Secondary labeling was with Alexa Fluor donkey anti-mouse 488 or donkey anti-rabbit 568. Nuclei were label with DAPI. Immunocytochemistry followed the above method excluded antigen retrieval steps. All fluorescence was imaged using an inverted fluorescence (Leica DMI 6000 B) or confocal microscope (Zeiss LSM 710 META).

### Colocalization calculations and proximity ligation assay

The colocalization of confocal immunofluorescence images was calculated using the WCIF ImageJ plugin (WCIF-ImageJ, UHN Research). NG2-type VI collagen colocalization experiments were performed using the MAB5384 (mouse origin) and 70R-CR009x (rabbit origin) primary antibodies. NG2-endocytosis colocalization experiments were performed using the MAB5320 primary antibody (rabbit origin) against CHC, AP2, and DNM2 antibodies of mouse origin. The amount of colocalization was quantified using a Mander’s Overlap Coefficient defined as the sum of the products of the two channels absolute intensities divided by the maximal product, corresponding to perfect colocalization. Colocalization is visually assessed with Product of the Difference from the Mean (PDM) plots. A sample size of three mice was used for colocalization calculations, with two slides stained from each mouse. A minimum of five cells was quantified from each cell layer in each slide. Colocalization was further tested using a Proximity Ligation Assay following the manufacturer protocol (Duolink, Sigma, DUO92101). In short, tissue samples were incubated with the NG2 mouse monoclonal (Millipore, MAB5384; clone 132.39) and Collagen 6α1 rabbit polyclonal (Fitzgerald, 70R-CR009x) antibodies, probes were added and hybridized, ligation was performed, and a fluorescent probe was added to indicate protein-protein interactions. Proximity ligation assay was only performed on tissue from non-surgical control mice with a sample size of three mice.

### NG2 Internalization

Internalization of NG2 was quantified from the NG2/type VI collagen confocal immunofluorescent images using the Analyze Particles plugin in ImageJ^40^. The cytosol of an individual cell was defined by a region of interest inside of the pericellular type VI collagen. NG2 colocalized with type VI collagen was excluded from the ROI. For the particle count, a standardized threshold of 30 and a minimum particle size of 5 pixel^2^ was used for all calculations. A sample size of three mice was used for colocalization calculations, with two slides stained from each mouse. A minimum of five cells were quantified from each tissue layer in each slide. The internalized particle count for each cell layer and across experimental groups was statistically compared using a two-sample t-test assuming unequal variances.

## Data Availability

All relevant data are within the paper.
